# Recovery of Waste Polyurethane from E-Waste—Part I: Investigation of the Oil Sorption Potential

**DOI:** 10.3390/ma14216230

**Published:** 2021-10-20

**Authors:** Vincenzo Santucci, Silvia Fiore

**Affiliations:** Department of Engineering for Environment, Land, and Infrastructures (DIATI), Politecnico di Torino, Corso Duca degli Abruzzi 24, 10129 Torino, Italy; vincenzo.santucci@polito.it

**Keywords:** absorption, circular economy, oil spill, refrigerator, WEEE

## Abstract

The shredding of end-of-life refrigerators produces every year in Italy 15,000 tons of waste polyurethane foam (PUF), usually destined for energy recovery. This work presents the results of the investigation of the oil sorption potential of waste PUF according to ASTM F726–17 standard. Three oils (diesel fuel and two commercial motor oils) having different densities (respectively, 0.83, 0.87, and 0.88 kg/dm^3^) and viscosities (respectively, 3, 95, and 140 mm^2^/s at 40 °C) were considered. The waste PUF was sampled in an Italian e-waste treatment plant, and its characterization showed 16.5 wt% particles below 0.71 mm and 13 wt% impurities (paper, plastic, aluminum foil), mostly having dimensions (d) above 5 mm. Sieving at 0.071 mm was applied to the waste PUF to obtain a “coarse” (d > 0.71 mm) and a “fine” fraction (d < 0.71 mm). Second sieving at 5 mm allowed an “intermediate” fraction to be obtained, with dimensions between 0.71 and 5 mm. The oil sorption tests involved the three fractions of waste PUF, and their performances were compared with two commercial oil sorbents (sepiolite and OKO-PUR). The results of the tests showed that the “fine” PUF was able to retain 7.1–10.3 g oil/g, the “intermediate” PUF, 4.2–7.4 g oil/g, and the “coarse” PUF, 4.5–7.0 g oil/g, while sepiolite and OKO-PUR performed worse (respectively, 1.3–1.6 and 3.3–5.3 g oil/g). In conclusion, compared with the actual management of waste PUF (100 wt% sent to energy recovery), the amount destined directly to energy recovery could be limited to 13 wt% (i.e., the impurities). The remaining 87 wt% could be diverted to reuse for oil sorption, and afterward directed to energy recovery, considered as a secondary option.

## 1. Introduction

Approximately 97,000 tons of waste temperature exchange equipment were collected in Italy in 2020, mostly consisting of end-of-life (EoL) refrigerators [1]. This type of waste falls into category 1 of waste electrical and electronic equipment (WEEE, or e-waste), as defined by the Directive 2012/19/EU [2]. On average in Italy 90 wt% of collected EoL temperature exchange equipment is made of refrigerators (the rest are air conditioners), whose 15–16 wt% is made of polyurethane foam (PUF) sprayed inside the frame as thermal-insulating material [3,4]. Hence, the expected amount of waste PUF deriving from EoL refrigeration equipment is nearly 15,000 tons every year. In the current Italian WEEE management system, waste PUF is often an unrecovered fraction, since there is no common practice to reuse it as secondary raw material, and it is disposed of in incineration plants. After the Paris agreement, signed in 2015 by members of the United Nations on the reduction in greenhouse gas (GHG) emissions, and the New Circular Economy Action Plan, stated in 2020 by the European Commission, WEEE management optimization become a strategic issue in urgent need of optimization. Therefore, research activity driven by the aims of reducing GHG emissions and keeping the resources in use as long as possible is highly needed. With the aim of exploring the potential perspectives for material recovery from waste PUF, the scientific and technical literature was surveyed according to the following two complementary points of view:

- *A top-down approach* considering the general uses of PUF: the main applications (as raw or secondary material) in the global manufacturing industry were identified. The most common applications of PUF are connected with two purposes: thermal insulation (in buildings and refrigeration equipment) due to its outstanding thermal properties [5,6,7], and the manufacturing of flexible and porous items such as mattresses, car seats, shoes, sports equipment, etc. due to the facility of molding it into the desired frame and/or applying it as spray foam [8,9].
- *A bottom-up approach* based on the uses of recovered waste PUF: the technical properties required for polyurethane for specific applications were evaluated. Three promising solutions for the valorization of waste PUF were found: application as sorbent material for oil spills [10,11,12]; additive for construction materials as lightweight mortars or insulant materials [13,14,15,16]; application in filters for the adsorption of pollutants from wastewater [17,18].

Matching the results of the literature survey based on the two complementary viewpoints, the research was focused on two main directions and organized into two parts: part I explores the oil sorption potential, which is presented in this work, and part II, investigates the adsorption potential toward inorganic and organic contaminants for wastewater treatment, which will be discussed in another work [19]. Part I of the research, here presented, specifically considers the application of PUF as contingency equipment for absorbing oil spills. Due to its interesting properties (high porosity, hydrophobicity), PUF is applied as the primary raw material in the manufacturing of industrial absorbents, which can absorb crude oil and related by-products. US EPA defines fundamental features for such products to be oleophilic (oil attracting) and hydrophobic (water repellent) [20]. Contingency equipment for oil spills in the form of pads and booms made of PUF can absorb 20–40 times their own weight in oil, whereas synthetic granular sorbents have generally lower capacity of absorption, between 5 and 30 times their weight [21,22]. Since the excessive use of sorbents at a spill scene, especially in granular or particulate forms, can lead to cleanup problems, their applications are intended for industrial workplaces such as warehouses and repair shops [23,24].

Many studies on PUF were devoted to the improvement of the performance of products deriving from primary materials, tested on oils and several waste fluids, through the chemical treatment of the porous surface, or applying special synthesis processes [25,26,27,28]. Recently, numerous scientific studies focused on how to recover waste PUF by chemical or thermochemical recycling processes [29,30,31,32,33], but, to our knowledge, there is not yet any consistent research about the absorbing performances of waste PUF that can be achieved without altering its chemical structure. This work aimed at defining an easily applicable valorization opportunity of waste PUF in loose form, based on physical/mechanical treatment processes commonly performed in a WEEE treatment plant. The experimental plan of the here-presented study was designed to assess the effectiveness of waste PUF derived from EoL refrigerators in absorbing oily substances. The purpose of this specific research is that, once the lifespan of the refrigerator is over, a new use phase for the PUF as recovered absorbent would be feasible, with the lowest possible requirements about technical complexity and related economic and environmental costs. Compared with recycling through chemical treatments, this approach can limit energy consumptions and the generation of residual by-products, thus resulting in an easier and cheaper technical solution. The outcomes of this research can provide support in the evaluation of material-recovery opportunities from WEEE consistently with circular economy principles, and in the optimization of the management of waste PUF from EoL refrigerators.

## 2. Materials and Methods

### 2.1. Waste Origin and Sampling

The tested material was PUF in loose form, derived from the mechanical treatment of EoL refrigerators (category 1 WEEE) at the TBD treatment plant, a few km from Turin, Italy, and managed by AMIAT SpA. AMIAT oversees municipal solid waste management in Turin (900,000 inhabitants). The catchment area of the TBD plant extends through northwestern Italy, accounting in total for 3300 t of category 1 WEEE in 2018. Several treatments were applied in the TBD plant on the EoL refrigerators: initial disassembling of the refrigerating circuit and depollution from hazardous fluids; shredding of the carcasses to recover ferrous and non-ferrous metals, respectively, through magnetic and eddy current separators, while mixed plastics are processed in another plant. The heterogeneous material resulting from the shredding phase underwent an air separation treatment in a “zig-zag” process, which diverted PUF to a milling phase to achieve particle size below 10 mm. Finally, the milled PUF was briquetted and sent to incineration.

The milled PUF was sampled for the research along 5 weeks, one sample per week, to account for any composition variability. The samples (1 kg each) were collected at the milling unit crate according to standard methods UNI 10802:2013 and UNI 14899:2006. The samples were assumed representative, considering that 3300 t/y EoL refrigerators entering the plant roughly correspond to over 300 items shredded per day (average weight of 1 item: 42 kg) [3]. The collected samples were quartered to obtain representative secondary samples (40–50 g) for the characterization.

### 2.2. Characterization

Within the project, part of the characterization was performed in Iren Group laboratories, analyzing (the reference methods are detailed in parentheses): the speed of combustion (UNI CEN/TS 16023:2014), the chemical composition (UNI EN 13657:2004 and UNI EN ISO 11885:2009), the density (ASTM D 5057-10), the content of organic compounds, PCBs and pesticides (EPA 3545A 2007 + EPA 8270E 2018, EPA 5021A 2014 + EPA 8260D 2018), and performing UNI EN 12457-2 leaching test to assess the release of contaminants into the environment. The list of measured parameters with the related values is in the Supplementary Materials Section. The rest of the characterization, consisting of the particle-size and component analyses, was performed in the Circular Economy Lab at DIATI, Politecnico di Torino. The particle-size analysis was performed through a Giuliani IG3/EXP siever equipped with 7 sieves of different mesh screens. All analyses were performed on aliquots of five different secondary samples.

### 2.3. Pre-Treatment

**Figure 1:**
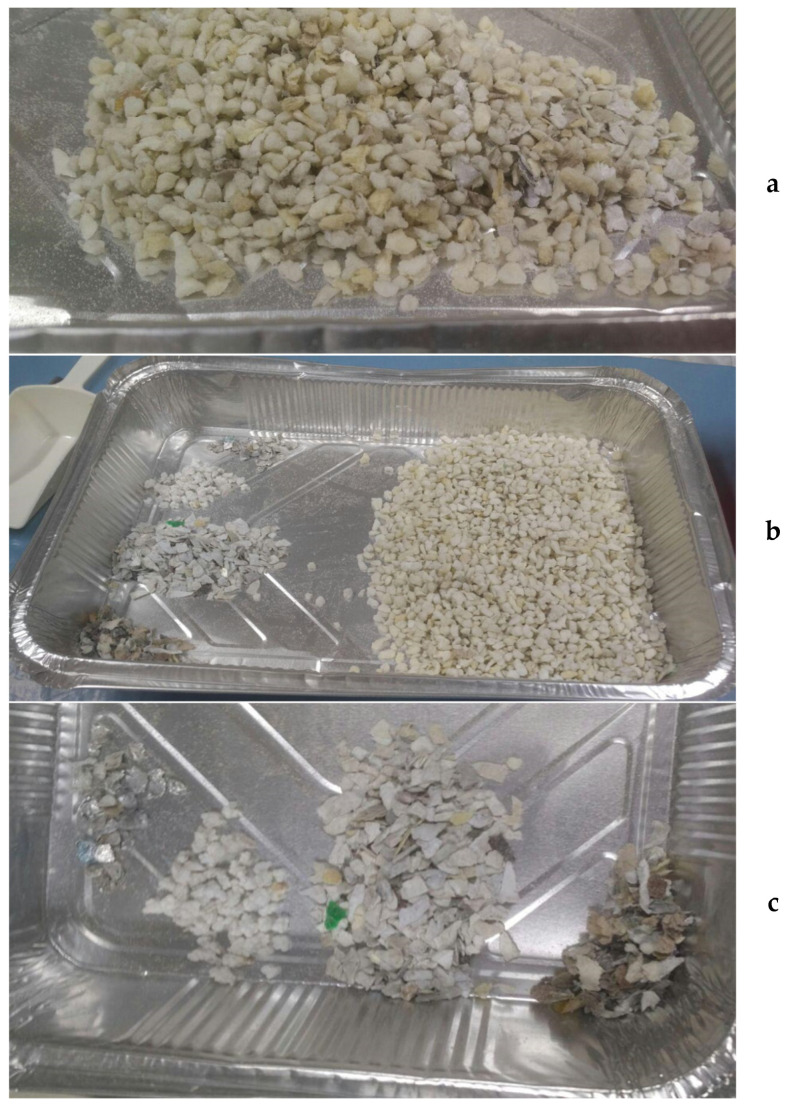
Appearance of the waste polyurethane (PUF) foam: (**a**) PUF sample collected at the milling unit; (**b**) PUF sample after the removal of impurities; (**c**) impurities separated by type.

The visual analysis of the waste PUF highlighted the presence of coarse impurities (plastic, paper, aluminum foil) (Figure 1) and of fine particles. Therefore, the samples underwent a sieving pre-treatment with 2 subsequent dimensional cuts (0.71 and 5 mm), aimed at separating the “fine” fraction (dimensions below 0.71 mm) from the “coarse” fraction (dimensions above 0.71 mm), and afterward, to eliminate the impurities (dimensions above 5 mm) and obtain an “intermediate” fraction, having dimensions between 0.71 and 5 mm (Table 1). The pre-treatment was performed on three different secondary samples (see Section 2.2).

### 2.4. Oil Sorption Tests

The tests were conducted on the three mentioned particle-size fractions of waste PUF deriving from the pre-treatment (Table 1). They were compared with two commercial products used as loose sorbents for oil spill control: sepiolite and OKO-PUR. Sepiolite is a traditional sorbent material made of clay minerals’ granules of magnesium silicate; OKO-PUR is a full saturation oil binder in powder, derived, likewise the tested PUF, from waste plastic foam. OKO-PUR and sepiolite were provided by Amiat and purchased from Airbank company (www.airbank.it, accessed on 8 May 2021). Their particle-size characteristics, as specified by the supplier, are presented in Table 1.

The tests were performed in triplicates according to the international ASTM F726–17 standard, which provides methods for assessing the performance of adsorbents in removing crude oils and related spills [34]. The PUF in loose form falls under the definition of Type II adsorbent in the ASTM F726–17 standard (“an unconsolidated, particulate material without sufficient form and strength to be handled except with scoops and similar equipment”). The oil sorption capacity was assessed as the ratio

(1) $$\frac{S_{O}}{S_{i}}\;$$

where

**Figure 2:**
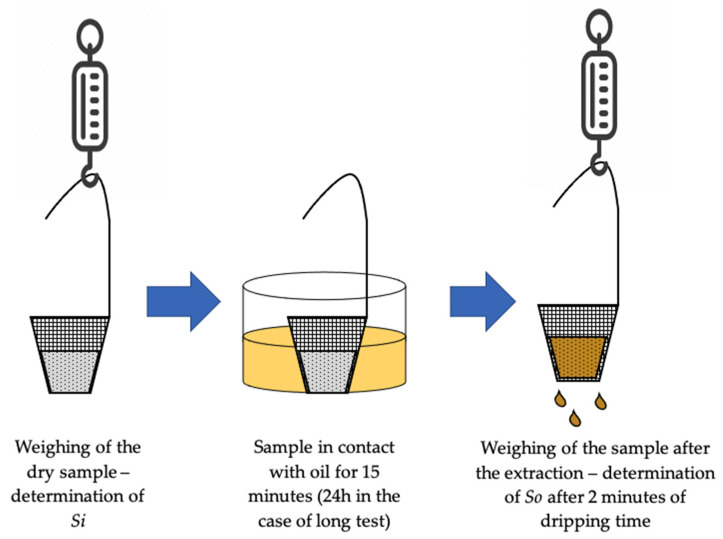
Scheme of the procedure applied for the oil sorption tests.

*S_O_* = *S_OT_* − *S_i_* (2)

is the net oil absorbed; $S_{OT}$ is the weight of the absorbent at the end of the test after 2 min dripping; $\;\;S_{i}$ is the initial dry absorbent weight (Figure 2). Specifically, the oil sorption capacity represents the amount of sorbed oil normalized by the absorbent’s mass. The oil sorption capacity of the three particle-size fractions of waste PUF and of the two commercial competitors was assessed toward three different oils, according to the ASTM F728-17 standard. Specifically, a diesel fuel (Quaser, produced by Q8) and two commercial motor oils (10w40 Prestige and 20w50 Select, produced by Delphi) with different densities (respectively 0.83, 0.87, and 0.88 kg/dm^3^) and viscosities (respectively 3, 95, and 140 mm^2^/s at 40 °C) were considered. The procedure for the sorption tests is detailed as follows: A 19 cm crystallizing dish was filled up to 2.5 cm height with the oil. The PUF sample (5 g) was placed in a steel mesh basket with 0.1 mm mesh openings and kept in contact with the oil for 15 min. During the contact, the gradual sinking of the whole absorbent mass was visible. Then, the sample was removed from the crystallizing dish, and the soaked absorbent with the mesh basket was drained for 10 min. Within this time, the oil in excess in contact with the absorbent’s surface, but not completely absorbed, is released by dripping. According to ASTM F726–17 standard, the weight measurement should be taken 30 s after removing the sample from the crystallizing dish. In case of heavy or weathered oils, a 2 min drain time is recommended. However, if the drainage is not adequately long, the values reported for the oil sorption capacity will be inaccurately high [35]. The weight measurements on the sample were taken after 0.5, 1, 2, 5, and 10 min to evaluate the actual amount of absorbed oil and the tendency to release it. In the case of the heaviest oil (20w50), preliminary tests showed that the sample was not fully saturated, since a part of it floated and did not sink after 15 min of contact with the oil. This behavior is typical of high-viscosity oils that require a longer time to saturate the pores of the tested material. This happened for the “intermediate” and OKO samples. Hence, a long test (24 h contact time) according to the ASTM F726-17 standard was conducted on these two materials.

## 3. Results and Discussion

### 3.1. Characterization

The results of the waste PUF characterization are reported in full detail in the Supplementary Materials Section. To summarize, the preliminary characterization revealed that the waste PUF in granular form had residual water content equal to zero, low bulk density (about 50 kg/m^3^), pH equal to 8, and 10 wt% ash. As expected, it was easily flammable, with LHV equal to 26,900 kJ/kg; this feature requires additional caution due to fire risk when storing and handling the material. Regarding this aspect, recent literature presents some studies on the synthesis of polyurethane with reduced flammability [36]. The chemical analyses highlighted the content of total carbon equal to 65.12%, and the presence of several metals (0.65% Al, 0.32% Fe, 0.23% Zn, 0.66% Ca), whereas for most organic compounds, PCBs, and pesticides, the concentrations were minimal (sum of aromatic compounds 1.95 mg/kg and hydrocarbons C1-C40 0.16%) or under the detection limit of the analytical methods (<0.1–0.2 mg/kg). The results of the UNI EN 12457-2 leaching test were compared with the maximum thresholds limits allowed by the Italian regulation for the recovery of secondary raw materials from non-hazardous waste [37]. Values above the thresholds were found for copper (0.14 mg/L compared with a 0.05 mg/L limit) and fluorides (55 mg/L compared with a 1.5 mg/L limit) only.

**Figure 3:**
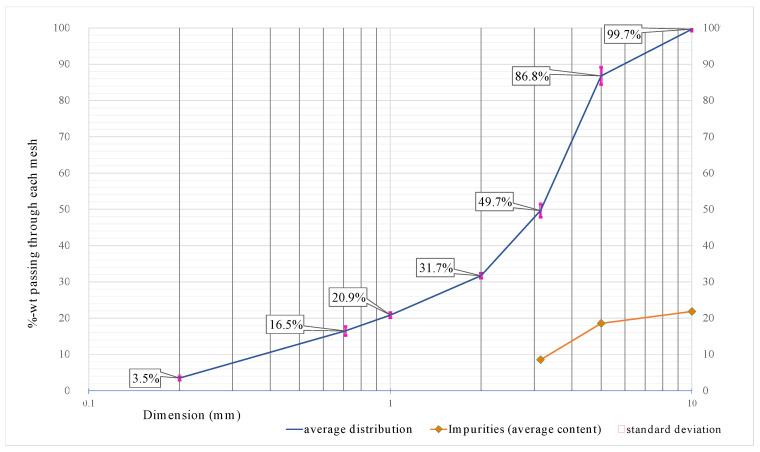
Particle-size distribution of the waste polyurethane foam.

The particle-size distribution of the waste PUF (Figure 3) showed that only 0.33 wt% of the sample had dimensions above 10 mm, and it was made of rigid plastic particles. The finest fraction (dimensions below 0.2 mm) was less than 3.5 wt%. The impurities (about the 25 wt% of waste PUF) were made of four main materials: rigid plastic (20 wt%), paper (3.6 wt%), aluminum (1.5 wt%), and polystyrene (0.4 wt%).

### 3.2. Pre-Treatment

The two-step sieving pre-treatment resulted in three particle-size fractions from the waste PUF: after the first sieving at 0.71 mm, a “fine” fraction, having dimensions below 0.71 mm (16.5 wt%) and a “coarse” fraction, having dimensions above 0.71 mm (83.5 wt%). After the second sieving at 5 mm to eliminate the impurities, the “intermediate” fraction having dimensions between 0.71 and 5 mm (70 wt%) was obtained.

### 3.3. Oil Sorption Tests

**Figure 4:**
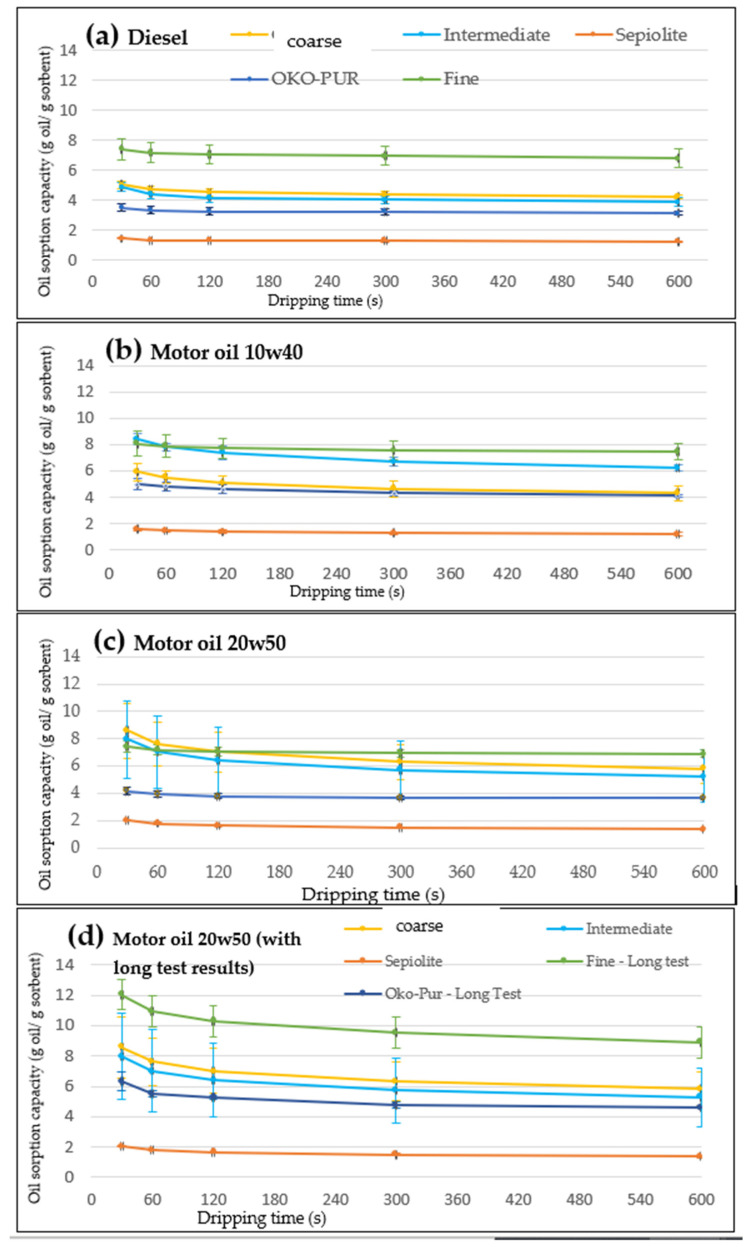
Results of the oil sorption capacity tests performed with (**a**) diesel, (**b**) 10w40 oil, (**c**) 20w50 oil, and (**d**) 20w50 oil in long test (contact time 24 h). The results obtained for the coarse PUF are indicated in the legend as “over 0.71”.

The sorption tests were performed on the fine and intermediate fractions obtained from the pre-treatments (Section 2.3 and Section 3.2), and on the two commercial products—Sepiolite and OKO-PUR. The results of the sorption tests (Figure 4) showed that for both commercial products the lowest sorption capacity was found with diesel fuel (Figure 4a), which has lower density compared with motor oils, which, in turn, directly resulted in lower oil sorption capacity. Sepiolite was characterized by oil sorption capacities between 1.3 and 2 g oil/g sorbent, depending on the type of oil. Similarly, OKO PUR showed oil sorption capacity ranges between 3.2 and 5.2 g oil/g sorbent. In these tests, since still an abundant oil release was observed at 30 s dripping time, the oil sorption capacity was calculated considering the weight of the sample after 2 min of dripping time. After this time interval, variations in oil sorption capacity were negligible, leading to more precise results. Hence, Table 2 reports the oil sorption capacities (relative to 2 min of dripping time) measured for the considered materials.

**Figure 5:**
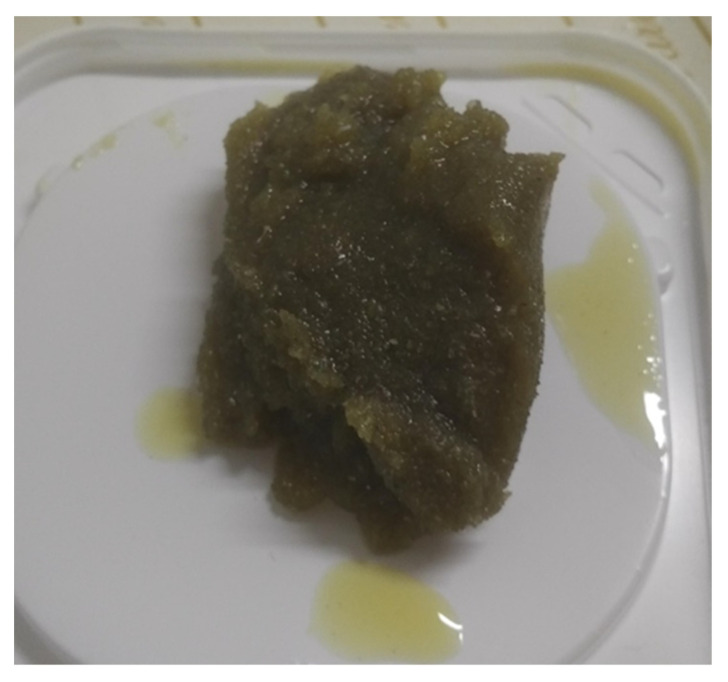
Fine sample of polyurethane foam soaked with 20w50 oil after oil sorption long test.

The best oil sorption performance (Table 2) was achieved by the fine fraction of the waste PUF, which was capable of adsorbing oil for over 7 times the sample weight in all tests. Considering the results of the short test, the highest oil sorption capacity (7.72 g oil/g sorbent) was achieved for the fine PUF with 10w40 motor oil. However, part of the fine PUF and OKO samples did not sink totally in contact with 20w50 oil, resulting in a significant gap in the saturation level of the absorbent. Considering the long test conducted for the fine PUF and OKO samples, the highest oil sorption capacity (10.30 g oil/g absorbent) was achieved for the fine PUF with 20w50 motor oil. Furthermore, the oil sorption capacity curves obtained from the fine PUF (Figure 4) showed that the release of oil after removing the absorbent from the crystallizing dish is significantly constrained. The curves related to the fine PUF are flatter in comparison with the ones obtained from the coarser fractions. One reason for this behavior can be the higher surface area of the fine PUF, which provides a higher number of sites in the micropores of the absorbent where the oil can create bonds. Indeed, it is reasonable to expect that the fine PUF has a higher surface area in comparison with the other samples. Additionally, a practical convenience of the fine powders, compared with coarser absorbents, is the physical state and compactness of the material soaked with oil. The fine PUF loose particles are bonded together by capillarity, resulting in a waste handy to collect after its application (Figure 5). After oil absorption, the PUF can be destined for thermal recovery. Compared with actual management (direct thermal recovery of 100% waste PUF), the scenario would change into 13 wt% waste PUF (i.e., coarse fraction) directly sent to thermal recovery, and 87 wt% reused as oil absorbent and afterward directed to thermal recovery as a secondary option.

The other particle-size fractions of the waste PUF were characterized by good oil sorption capacities, with oil sorption values higher for the motor oils than the diesel fuel (Table 2). Specifically, the values achieved were, respectively, 4.51, 5.11, and 7.02 g oil/g for the coarse PUF, and 4.17, 7.36, and 6.41 for the intermediate PUF. These results, considered in comparison with the ones achieved for fine PUF, highlighted a clear improvement in the oil sorption performance when coarser fractions were removed from the waste PUF. Finally, considering the commercial oil sorbents, OKO-PUR demonstrated performances (3.26–5.27 g oil/g) that can be considered comparable with the coarse and intermediate PUF, while sepiolite sorption capacity was worse (1.3–1.6 g oil/g).

To better understand whether the oil sorption capacities of tested PUF samples are satisfactory, the results from similar experimental studies conducted on both commercial and alternative (i.e., waste-derived) materials were compared in Table 3. The absorbent materials derived from recovered wastes, classified in Table 3 according to their origin (industrial, mineral, organic/vegetable), were characterized by oil sorption capacities between 3 and 28.5 g of oil/g of absorbent. Commercial sorbents, instead, were characterized by oil sorption capacities between 3 and 31 g of oil/g of absorbent. Therefore, the performances achieved in this work by waste PUF can be assumed consistent with the literature.

## 4. Conclusions

The idea at the base of this work was to obtain, in the same WEEE treatment plant producing the waste PUF, secondary PUF ready for a new life, which could be then considered a *by-product* according to EU regulations (e.g., “destined for a use for which there is a market and without any harm for the environment and human health, after the application of processes common in the industrial practice”) instead of a waste. A simple sieving process performed on waste PUF with two-dimensional cuts (0.71 and 5 mm) allowed the impurities to be separated (dimensions above 5 mm, directly sent to thermal recovery), while the rest could be diverted to reuse as an oil absorbent. The oil sorption performances of waste PUF were promising, compared with commercial mineral (sepiolite) and organic (OKO-PUR) products commonly used to control oil spills. Particularly, the fine fraction of waste PUF (dimensions below 0.71 mm) revealed oil sorption performances at least 2–3 times higher than the commercial products. The performances achieved for waste PUF in this work can be assumed interesting also considering the literature data on commercial and alternative absorbents. This work may pave the way for further research in the field, which is needed to develop “end-of-waste” guidelines for waste PUF, e.g., the operations and requirements defined to convert waste PUF into a secondary raw material according to EU regulations. The approach of this work was consistent with circular economy principles in a difficult sector as WEEE recycling and recovery and allowed to demonstrate that material recovery can be possible for waste PUF and that thermal recovery could be a secondary option.

## Figures and Tables

**Table 1:** Features of different types of samples used in oil sorption tests.

Size Range (mm)	Sample ID	Bulk Density (kg/m^3^)
d < 0.71	fine	129.65
0.71< d < 5	intermediate	47.57
d > 0.71	coarse	42.00
2 < d < 4	sepiolite	440
d < 1	OKO-PUR	127.5

**Table 2:** Oil sorption capacities of the tested materials (values expressed in g of oil/g of sorbent). The results are expressed as mean of three replicates with related standard deviation.

Sample Description	Diesel Fuel	Motor Oil 10w40	Motor Oil 20w50
PUF coarse (d > 0.71 mm)	4.51 ± 0.64	5.11± 0.50	7.02 ± 1.49
PUF intermediate (0.71 < d < 5 mm)	4.17 ± 0.31	7.36 ± 0.50	6.41 ± 2.41
PUF fine (<0.71 mm)	7.07 ± 0.23	7.72 ± 0.80	10.30 * ± 0.89 *
OKO-PUR	3.26 ± 0.24	4.60 ± 0.30	5.27 * ± 0.11 *
Sepiolite	1.30 ± 0.01	1.34 ± 0.14	1.64 ± 0.06

* long test (24 h contact time).

**Table 3:** Oil sorption capacities reported in the literature for commercial and alternative absorbent materials.

Absorbent Type	Description	Oil Sorption Capacity (g/g), by Short Test	Oil Sorption Capacity (g/g) by Long Test	Dripping Time (s)	Pre-Treatment	Source
Commercial organic sorbents	Reo Amos. Commercial oil sorbent, mixture of loose sorbents in a polypropylene matrix (0.5–5 mm)	Oil 10w40 9.24	-	30	Hydrophobic treatment	[21]
	ACME cellular synthetic sorbent	Diesel 5.8 Medium-density oil 12.3 High-density oil 9.7	Diesel 6.2 Medium-density oil 12 High-density oil 14.3	30 120 for heavy oil	Not available	[38]
	Osprey cellulose-based sorbent	Diesel 18.06 Medium-density oil 21.85 High-density oil 29.96	Diesel 18.11 medium-density 24.60 high-density oil 30.66	30	Not available	[39]
Commercial mineral sorbents	Expanded perlite, from amorphous aluminous silicate (0.5–2.5 mm)	Oil 10w40 3.33	-	30	Industrial treatments	[21]
	Absodan Plus, made from clay minerals (1.5–3 mm)	Oil 10w40 1.06	-	30	Industrial treatments	[21]
	Eco-dry plus, from mixture of rock-based minerals (0.5–2.5 mm)	Oil 10w40 1.37	-	30	Industrial treatments	[21]
Industrial waste-derived sorbents	Aerogels from waste-tire derived textile fibers (recycled)	Oil 5W-30 10.3 (2 h of contact)		300 s	Chemical and physical treatment	[40]
	Carton and paper scraps	Diesel 9.6 Oil 0w30 12 Oil 10w30 12	-	30	Surface modification, chemical	[41]
Organic sorbents at experimental level	Microplastics (size 8.6 μm)	Crude oil 1.73 (10 min of contact)	-	60	Sample inserted in an envelope made of polypropylene	[42]
	Polyurethane foam	Diesel 8 Gasoline 18	-	60	Surface modification, chemical	[20]
Sorbents derived from mineral waste	Generic soil (0.1–4 mm)	Oil 10w40 0.45–3.82	-	30	Drying	[21]
Sorbents derived from organic and vegetable waste	Sugarcane bagasse (average size 0.2 mm)	Crude oil 3.3–8	-	30	Comminution, Drying	[43]
	Fibers from phragmites australis	Crude oil 5.5	-	30	Comminution, Drying	[43]
	Sugarcane leaves straw	Crude oil 4.5	-	30	Comminution, Drying	[43]
	Switchgrass (average particles size mm)	Oil 10W-30 (2 h of contact) 3.0	-	1800	Grounding	[44]
	Needles from larch, fir, and pine trees	Oil 10w40 4.5	-	30	Drying	[21]
	Beech sawdust	Oil 10w40 7.01	-	30	Drying	[21]
	Spruce sawdust	Oil 10w40 6.54	-	30	Drying	[21]
	Leaf residues	Oil 10w40 15.47	-	30	Drying	[21]
	Moss	Oil 10w40 28.47	-	30	Drying	[21]
	Coconut coir	Vegetable oil 7.2 Diesel fuel 6.5	-	30	Drying	[45]
	Banana peels (average particle size 0.36 mm)	Diesel 6.8 Vegetable oil 7.2	-	30	Comminution, Drying	[46]

## Supplementary Materials

The following are available online at https://www.mdpi.com/article/10.3390/ma14216230/s1, Table S1: Characterization of the waste PUF.
