# Supplementary material for: Recovery of Waste Polyurethane from E-Waste—Part I: Investigation of the Oil Sorption Potential
Source: Materials (Basel). 2021 Oct 20;14(21):6230. doi: 10.3390/ma14216230 (PMC8584342; doi:10.3390/ma14216230)
Supplement: Supplementary file 1 [file materials-14-06230-s001.zip › materials-1355312-supplementary.pdf]

Supplementary Material

Table S1. Results of waste PUF characterization. Values expressed as average of 5 replicates.

parameter	measure unit	value	standard deviation
Identification of the substance		dust/granular/loose	
Speed of combustion test	s	42.73	1.26
Outcome of the test		easily flammable	
LHV	kJ/kg	26900.00	2051.34
Dry substance	%	100.00	0
Ashes at 550 °C	% dry substance	10.40	1.60
pH in water	pH units	8.02	0.16
Density	kg/dm^3	0.05	0.01
Total Carbon	%	65.12	3.12
Ammonia	mg/kg	< 2.5	-
Aluminum	mg/kg	6537.60	3614.21
Antimony	mg/kg	179.60	326.33
Arsenic	mg/kg	< 10	-
Berillium	mg/kg	< 10	-
Barium	mg/kg	152.00	28.20
Boron	mg/kg	< 10	
Cadmium	mg/kg	< 10	-
Cobalt	mg/kg	< 10	-
total Chromium	mg/kg	39.00	12.52
Iron	mg/kg	3182.20	670.11
Phosphorus	mg/kg	106.80	22.11
Manganese	mg/kg	22.20	4.71
Mercury	mg/kg	< 0.1	-
Molibdenum	mg/kg	< 10	-

Nickel	mg/kg	12.25	4.91
Lead	mg/kg	43.00	35.42
Copper	mg/kg	385.80	346.12
Selenium	mg/kg	< 10	-
Tin	mg/kg	12.00	1.87
Tallium	mg/kg	< 10	1.87
Tellurium	mg/kg	< 10	-
Vanadum	mg/kg	< 10	-
Zinc	mg/kg	2284.80	668.46
Calcium	mg/kg	6616.00	1985.44
Magnesium	mg/kg	334.20	228.54
Potassium	mg/kg	552.20	115.76
Sodium	mg/kg	196.00	30.59
Benzo(a)antracene	mg/kg	< 0.1	-
Benzo(a)pyrene	mg/kg	< 0.1	-
Benzo(e)pyrene	mg/kg	< 0.1	-
Benzo(b)fluoranthene	mg/kg	< 0.1	-
Benzo(k)fluoranthene	mg/kg	< 0.1	-
Benzo(j)fluoranthene	mg/kg	< 0.1	-
Benzo(g.h.i)perylene	mg/kg	< 0.1	-
sum Benzo(b. j. k)fluoranthene	mg/kg	< 0.1	-
Crysene	mg/kg	< 0.1	-
Dibenzo(a.e)pyrene	mg/kg	< 0.1	-
Dibenzo(a.l)pyrene	mg/kg	< 0.1	-
Dibenzo(a.i)pyrene	mg/kg	< 0.1	-
Dibenzo(a.h)pyrene	mg/kg	< 0.1	-
Dibenzo(a.h)anthracene	mg/kg	< 0.1	-
Indeno(1.2.3-c.d)pyrene	mg/kg	< 0.1	-
Pyrene	mg/kg	< 0.1	-

Sum of polycyclic aromatic	mg/kg	< 0.1	-
Cumene (isopropylbenzene)	mg/kg	< 0.2	-
Dipenthene	mg/kg	< 0.2	-
Naftalene	mg/kg	< 0.1	-
Anthracene	mg/kg	< 0.1	-
Fluoranthene	mg/kg	< 0.1	-
Acenaftilene	mg/kg	< 0.1	-
Acenafthene	mg/kg	< 0.1	-
Fluorene	mg/kg	< 0.1	-
Fenanthrene	mg/kg	< 0.1	-
Benzene	mg/kg	< 0.1	-
1.3 Butadiene	mg/kg	< 0.1	-
Etilbenzene	mg/kg	< 0.1	-
Styrene	mg/kg	1.77	0.76
Toluene	mg/kg	0.18	0.36
m.p-xylene	mg/kg	< 0.1	-
o-xylene	mg/kg	< 0.1	-
sum o.m.p-xylene	mg/kg	< 0.1	-
Sum of aromatic organic compounds (excluding Benzene)	mg/kg	1.95	1.04
Chloromethane	mg/kg	< 0.1	-
Dichloromethane	mg/kg	< 0.1	-
Trichloromethane	mg/kg	< 0.1	-
Vinyl chloride	mg/kg	< 0.1	-
1.2-dichloroethane	mg/kg	< 0.1	-
Trichloroethylene	mg/kg	< 0.1	-
Tetrachloroethylene	mg/kg	< 0.1	-
1.1-dichloroethane	mg/kg	< 0.1	-
cis 1.2-dichloroethylene	mg/kg	< 0.1	-

trans 1.2-dichloroethylene	mg/kg	< 0.1	-
sommatoria cis-trans 1.2-dichloroethylene	mg/kg	< 0.1	-
1.1.1-trichloroethane	mg/kg	< 0.1	-
1.2-dichloropropane	mg/kg	< 0.1	-
1.1.2-trichloroethane	mg/kg	< 0.1	-
1.2.3 Trichloropropane	mg/kg	< 0.1	-
1.1.2.2 tetrachloroethane	mg/kg	< 0.1	-
Tribromomethane	mg/kg	< 0.1	-
1.2-dibromoethane	mg/kg	< 0.1	-
Dibromochloromethane	mg/kg	< 0.1	-
Bromodichloromethane	mg/kg	< 0.1	-
Esachlorobutadiene	mg/kg	< 1	-
1.2-dichlorobenzene	mg/kg	< 0.1	-
1.4-dichlorobenzene	mg/kg	< 0.1	-
1.2.4-trichlorobenzene	mg/kg	< 0.1	-
1.2.4.5-tetrachlorobenzene	mg/kg	< 0.1	-
Esachlorobenzene	mg/kg	< 0.1	-
Pentachlorobenzene	mg/kg	< 0.1	-
Pentachlorobenzene	mg/kg	< 0.1	-
Alaclor	mg/kg	< 0.1	-
Aldrin	mg/kg	< 0.1	-
alfa-esachlorocycloesane	mg/kg	< 0.1	-
beta-esachlorocycloesane	mg/kg	< 0.1	-
cis-chlordane	mg/kg	< 0.1	-
gamma-esaclorocicloesano (lindane)	mg/kg	< 0.1	-
Clordecone	mg/kg	< 0.1	-
Mirex	mg/kg	< 0.1	-
Toxafene	mg/kg	< 0.1	-

DDD	mg/kg	< 0.1	-
DDE	mg/kg	< 0.1	-
DDT	mg/kg	< 0.1	-
Dieldrin	mg/Kg	< 0.1	-
Endrin	mg/kg	< 0.1	-
Demeton	mg/kg	< 0.1	-
Diazinon	mg/kg	< 0.1	-
Disulfoton	mg/kg	< 0.1	-
Ethion	mg/kg	< 0.1	-
Guthion	mg/kg	< 0.1	-
Malathion	mg/kg	< 0.1	-
Parathion ethyl	mg/kg	< 0.1	-
Parathion methyl	mg/kg	< 0.1	-
Aliphatic hydrocarbons C5-C8	mg/kg	1.24	0.78
Hydrocarbons C10-C40	mg/kg	1568.4	1415.76
Eptachlor epoxyde	mg/kg	< 0.1	-
Endosulfan sulphate	mg/kg	< 0.1	-
Esabromobiphenyl	mg/kg	< 0.1	-
"dioxin like" PCBs congeners			
#77	mg/kg	< 1	-
#81	mg/kg	< 1	-
#105	mg/kg	< 1	-
#114	mg/kg	< 1	-
#118	mg/kg	< 1	-
#123	mg/kg	< 1	-
#126	mg/kg	< 1	-
#156	mg/kg	< 1	-
#157	mg/kg	< 1	-

#167	mg/kg	< 1	-	
#169	mg/kg	< 1	-	
#189	mg/kg	< 1	-	
PCBs congeners				
#28	mg/kg	< 1	-	
#52	mg/kg	< 1	-	
#95	mg/kg	< 1	-	
#99	mg/kg	< 1	-	
#110	mg/kg	< 1	-	
#101	mg/kg	< 1	-	
#128	mg/kg	< 1	-	
#138	mg/kg	< 1	-	
#146	mg/kg	< 1	-	
#149	mg/kg	< 1	-	
#170	mg/kg	< 1	-	
#151	mg/kg	< 1	-	
#153	mg/kg	< 1	-	
#177	mg/kg	< 1	-	
#183	mg/kg	< 1	-	
#180	mg/kg	< 1	-	
#187	mg/kg	< 1	-	
**Results of the leaching test UNI EN 12457-2:2004**				**Limits for the recovery of non hazardous waste [33]**
pH	pH units	8.52		5.5-12
Conductivity	µS/cm	282.60	314.41	-
Antimony	mg/l	0.01	0.01	-
Arsenic	mg/l	< 0.03	-	0.05
Barium	mg/l	0.18	0.02	1
Cadmium	mg/l	< 0.002	-	0.005

Total Chromium	mg/l	0.06	0.03	50
Mercury	mg/l	< 0.0012	-	0.001
Molibdenum	mg/l	< 0.06	-	-
Nickel	mg/l	< 0.06	-	0.01
Lead	mg/l	0.04	0.04	0.05
Copper	mg/l	0.14	0.01	0.05
Boron	mg/l	0.10	0.01	-
Selenium	mg/l	< 0.002	-	0.010
Zinc	mg/l	0.75	0.24	3
Dissolved Organic Carbon (DOC)	mg/l	27.20	29.84	30
Fluoride	mg/l	55.00	-	1.5
Chlorides	mg/l	37.66	57.18	100
Sulphates	mg/l	13.84	13.06	250
TDS (total dissolved solids)	mg/l	232.00	191.56	-
Halogenated organic compounds measurd as Chlorine	% t.q.	0.77	0.24	-
Total Fluorine	% t.q.	0.04	-	-
Total Chlorine	% t.q.	0.94	0.28	-
Total Bromine	% t.q.	0.04	0.03	-
Total Iodine	% t.q.	0.01	-	-
Total Sulphur	mg/kg	123.00	25.98	-
